# Bioprospecting and Molecular Identification of Used Transformer Oil-Degrading Bacteria for Bioplastics Production

**DOI:** 10.3390/microorganisms10030583

**Published:** 2022-03-08

**Authors:** Shehu Idris, Rashidah Abdul Rahim, Al-Ashraf Abdullah Amirul

**Affiliations:** 1 School of Biological Sciences, Universiti Sains Malaysia, Gelugor 11800, Penang, Malaysia; idris.shehu@kasu.edu.ng (S.I.); rshidah@usm.my (R.A.R.); 2 Department of Microbiology, Kaduna State University, Kaduna PMB 2339, Nigeria; 3 Centre for Chemical Biology, Universiti Sains Malaysia, Bayan Lepas 11900, Penang, Malaysia

**Keywords:** bioprospecting, waste transformer oil, bioplastic, polyhydroxyalkanoates

## Abstract

One of the major impediments to the commercialization of biodegradable plastic is the high cost of substrate. Consequently, there is a continuous search for effective microorganisms and cheaper carbon substrates to reduce the high production cost. In this study, waste transformer oil-degrading bacteria were isolated from soil, wastewater, and sediment samples, using a mineral salt medium (MSM) supplemented with 1% waste transformer oil as the sole carbon source. The isolates were screened for polyhydroxyalkanoates (PHA) production using Nile red staining and fluorescence microscopy. PHA granules accumulation was confirmed using transmission electron microscopy. Oil degradation analysis was accomplished using solvent extraction and gravimetric methods whereas, the bacteria were identified using 16S DNA sequence homology. A total of 62 transformer oil-degrading bacteria were isolated, out of which 16 (26%) showed positive results for Nile red fluorescence microscopy. The identified organisms belong to four different taxonomic genera of *Acinetobacter*, *Bacillus*, *Proteus*, and *Serratia*. The percentage of oil degradation observed among the different isolates ranged between 19.58% and 57.51%. Analysis of the PHA extracted from the selected isolate revealed the presence of medium chain length polyhydroxyalkanoates (mcl-PHA). The findings of this work have further highlighted the diversity of the bacteria capable of utilizing waste streams such as waste transformer oil. Consequently, the isolates can be explored as agents of converting waste transformer oil into bioplastics.

## 1. Introduction

Synthetic plastics have become an important commodity considered to have improved the quality of human life, replacing packaging materials like glasses and paper [1]. Rapid development in material science and technology has undoubtedly brought about new plastic products with excellent mechanical integrity as well as durability [2]. However, the production of synthetic plastics has a great disadvantage of creating non-degradable waste products that are difficult to handle. The non-biodegradable property of these products, particularly high molecular mass and complex structure, impart a serious environmental problem as they can persist in the soil, water bodies, and landfills. Consequently, the accumulation of plastic waste in the environment has become a source of major concern and a worldwide issue [1]. The current situation of waste plastics management in developed nations, though slowly improving, is far from satisfactory. As for the developing countries, such waste management can be described as less promising [3]. Several researchers across the globe have been trying to provide a solution to this ever-increasing environmental challenge.

Over a long period, petroleum-based mineral oils have been used in electrical transformers, primarily for insulating purposes; the oil also serves as a coolant for the transformers. However, the long-term usage of transformer oil results in some changes in its physical and chemical characteristics, which consequently makes it unfit for cooling and insulating purpose [4]. Lack of proper storage and disposal of the used oils, as well as explosions of transformers, can cause a serious environmental pollution problem. Thus, the disposal of used transformer oil (UTO) from electrical power stations, as well as a large number of electrical transformers located in populated areas and shopping centers throughout the world, is becoming increasingly complex. This is because it could contaminate waterways and soil if serious spills happen. This problem necessitates the need to look for better management approaches to provide an immediate solution [4]. Coincidentally, there is a concerted effort in the search for cheaper substrates to produce biodegradable plastics to reduce the existing high production cost that remain a big challenge to the commercialization of this eco-friendly alternative product. From a technological viewpoint, polyhydroxyalkanoates (PHAs) draw much attention due to their biodegradability, thermoplasticity, and biocompatibility, as well as their production, which starts from abundantly available renewable resources [5]. PHAs seem to be potential candidates that could replace some conventional plastics [6].

According to recent reports, there have been several attempts to produce bioplastic using oils and waste oils, such as waste glycerol [7,8], palm oil [9], crude glycerol [10,11], and plant oils [12]. Reports on plastic consumption per capita indicate high demand. Therefore, it is evident that consumption of plastic products will be very difficult to reduce, probably due to the versatile properties of synthetic plastics and the corresponding low market price. However, there is a possibility of replacing petroleum-based plastics with alternative materials with similar properties that degrade after being discarded [2]. PHAs are a family of polyesters having side chains that differ in length from a simple methyl moiety (Polyhydroxybutyrate) to alkyl chains up to more than 15 carbons long. They are broadly classified into two divisions: namely, short-side chain length (*scl*) with up to two carbons in the side chain and medium-chain length (*mcl*) having three or more carbons in the side chain [13]. Those having 16 and above carbons in their monomers are considered long-chain length (*lcl*). Bacteria and other microbes can produce PHAs as intracellular carbon and energy reserves under stress conditions [14]. Although these bioplastics are biodegradable and bio-compatible, they are presently not priced competitive largely since the sugars (glucose) commonly used as fermentation feedstocks are expensive. Therefore, the importance of finding a less expensive substrate cannot be overemphasized [15].

Earlier applications were mostly in the areas of packaging (e.g., shampoo bottles, other cosmetic containers [16,17], covers for cardboards and papers, milk cartons and films, moisture barriers in nappies and sanitary towels, pens, combs, bullets, and bulk chemical production [18]. Microorganisms, particularly bacteria, can produce bioplastic from numerous carbon sources that include but are not limited to inexpensive, complex waste effluents, fatty acids, plant oils, alkanes, and simple carbohydrates [19]. UTO has been valorized for many purposes, especially biofuel [20] and alternative fuel [4,21]. Although the waste transformer oil has been the subject of many studies in terms of microbial degradation, to the best of our knowledge, waste oil has not been studied as a substrate for the production of PHAs. In this report, we described the isolation and screening of PHA-producing strains of the waste transformer oil-degrading bacteria to target the utilization of such organisms in the bioconversion of UTO into biodegradable plastics.

## 2. Materials and Methods

### 2.1. Collection of Samples

A total of 12 samples represented by soil, wastewater, and sediment were collected aseptically from various locations in Penang, Malaysia. The sampling sites include Pulau Burung, Sungai Chenaam, Sungai Pinang, Sungai Jelutong, Penang Bridge, and Batu Ferringi Beach. The samples collected were then transferred immediately to the laboratory at the School of Biological Sciences, Universiti Sains Malaysia, for analysis. All the samples were kept at 4 °C until required for use. The sample of used transformer oil was obtained from Tenaga National Berhad Research (TNBR) Malaysia.

### 2.2. Bacterial Isolation

The used transformer oil-degrading bacteria were isolated from the environmental samples via enrichment culture techniques. The isolation was accomplished using a mineral salt medium (MSM) supplemented with 1% v/v waste transformer oil as the sole carbon source. The basic medium (MSM) contains gram per liter of K2HPO4 (5.8); KH2PO4 (3.7); (NH4)2SO4 (1.1); MgSO4 7H2O (0.2). Other components include 1.0 mL of micro-element solution containing gram per liter FeSO4·7H2O (2.78); CaCl2·2H2O (1.67); MnCl2·4H2O (1.98); CoSO4·7H2O (2.81); CuCl2·2H2O (0.17); ZnSO4·2H2O (0.29); in 0.1 M HCl [22]. The used oil added to the medium was initially emulsified with one volume of Tween 80. One gram (1 g) of the solid samples (soil/sediment) or 1 mL, in the case of liquid samples (wastewater), was aseptically added to 100 mL MSM in a 250 mL Erlenmeyer flask. The culture was then incubated at 30 °C, 200 rpm for 72 h. Serial dilution of the enrichment culture was performed, followed by spread plate inoculation of the aliquot on the mineral salt agar containing 1% v/v used transformer oil. The inoculated culture plates were then incubated at 30 °C for 72 h. The morphologically distinct colonies were sub-cultured on the nutrient agar to obtain pure cultures of the isolates. Stock cultures were preserved in 20% glycerol and kept at −20 °C for the subsequent analysis.

### 2.3. Screening for PHA Production

The preserved isolates from the glycerol stock were transferred in 30 mL nutrient-rich broth (10 g/L peptone, 2 g/L yeast extract, and 10 g/L Lab Lemco) and grown at 30 °C, 150 rpm for 12–24 h. Subsequently, an aliquot (1 mL) taken from the culture was serially diluted in sterile distilled water. After that, 0.1 mL was taken from a selected dilution tube and inoculated on solid mineral salts medium containing 5 µg/mL Nile red [23]. The culture plates were then incubated at 30 °C for 24–48 h. Colonies that formed were sub-cultured in replicate onto a fresh medium. All the original plates were then exposed to ultraviolet illumination (320 nm) to identify PHA producers that exhibited pink fluorescence [22]. Fluorescence microscopic technique was also used to further identify the PHAs producing isolates. Briefly, 1 mL of the 72 h culture grown in MSM supplemented with 1% used transformer oil was transferred into the Eppendorf tube. About 50 μL of Nile red (5 μg/mL) and 500 μL distilled water were added to the cell suspension and Vortex mixer immediately. The suspension was kept at room temperature for 1–2 h and then centrifuged at 1000× *g* for 5 min. The supernatant was then discarded, and the pellet was washed twice with distilled water. About 500 μL of distilled water was then added to the pellet and vortex mix. After that, 10 μL of the stained cell suspension was placed onto a clean glass slide and covered with a glass slip. The edge of the glass slip covering the stained cells was then sealed with Cutex. Finally, the prepared samples were observed using a compound fluorescence light microscope (Olympus BX53, Olympus Optical Co. Ltd., Tokyo, Japan) equipped with an Olympus DP72 camera. The observation was completed under X100 UV compatible objective to confirm the presence of PHA granules [24]. Other microscopic analyses (SEM and phase-contrast microscopy) were executed as described elsewhere [9,25].

### 2.4. Analysis of Used Transformer Oil Degradation

The analysis of the used transformer oil degradation by the bacteria was performed using the gravimetric method after fermentation in 50 mL of MSM containing 2% *v*/*v* UTO, incubated at 30 °C for 72 h. In this technique, the residual oil in the culture supernatant was extracted for weight measurement to quantify the amount of the oil consumed during incubation. A solvent extraction method using n-hexane was adopted [26]. The weight of the used oil added into the medium (W1) was initially measured. After incubation, the culture supernatant containing the residual oil was recovered by centrifugation at 10,000× *g* for 5 min. About 5 mL of the solvent (n-hexane) was added to the supernatant in a clean 50 mL falcon tube and Vortex mixer. It was then transferred into a thistle funnel and kept for at least 4 h to allow for phase separation. The top layer (organic phase) containing a mixture of n-hexane and the residual oil was then separated from the aqueous layer. The aqueous phase was again mixed with an additional 5 mL n-hexane to extract the residual oil further. The total organic phase was subsequently collected into a pre-weighed Petri dish and kept overnight at room temperature for complete evaporation of n-hexane. The test was performed in triplicate, from which the mean was computed to minimize error. The gravimetric estimation of the percentage of the oil degradation was performed according to Equation (1) below:

Degradation (%) = “W_2_ − W_3_”/W_1_ × 100 (1)

where:

W_1_ = weight of the oil added to the medium.

W_2_ = weight of the petri dish plus weight of residual oil after solvent evaporation.

W_3_ = weight of the petri dish.

### 2.5. Shake Flask Fermentation and PHA Quantification

PHAs biosynthesis in the shake flask was performed as described elsewhere [22]. Briefly, the seed culture of each of the isolates was prepared in a nutrient-rich medium (10 g/L peptone, 2 g/L yeast extract, and 10 g/L Lab Lemco) and incubated at 30 °C and 150 rpm for 18 h. The MSM was inoculated with 0.6 g/L of the seed culture and incubated at 30 °C and 200 rpm for 72 h. The cells were harvested by centrifugation at 10,000× *g* for 10 min. The pellet was initially washed with distilled water and n-hexane to remove the traces of the residual oil before the final washing with distilled water. The pellet was then freeze-dried for subsequent analysis. To ascertain the presence and composition of the polymer, the isolated PHA or lyophilized cells were treated with H_2_SO_4_-methanol (85/15 [vol/vol]) in chloroform as previously described [27]. The dried cells (15–20 mg) were suspended in 2 mL chloroform in a test tube, 1 vol of the methanolysis solution (85:15 methanol: H_2_SO_4_) was added, and the tube was tightly capped. The suspension was heated at 100 °C for 140 min. The tubes were gently agitated at an interval of 20 min to facilitate the derivatization of the methyl ester of the polymer produced. After that, 1 mL of distilled water was added to the solution when it cooled to room temperature to aid the phase separation. The lower phase (about 1 mL) was carefully withdrawn using a Pasteur pipette and transferred into a clean bottle containing anhydrous sodium sulfate to dry any traces of water. A half milliliter (500 µL) of this solution was mixed with 1 vol of Caprylic methyl ester (internal standard) in a GC vial. The samples were then analyzed using a gas chromatography machine (GC-2014, Shimadzu, Kyoto, Japan).

### 2.6. PHAs Monomers Analysis

The PHA monomers were identified using gas chromatography-mass spectrometry (GC/MS), and nuclear magnetic resonance (NMR) as described else where [28]. The electron ionization mass spectra generated from the sample were automatically compared with the NIST08 library data. For the NMR analysis, a sample of the polymer was dissolved in deuterated chloroform to a final concentration of 25 mg/mL. The ^13^C nuclear magnetic resonance spectra of the polymer were recorded using an Ultrashield spectrometer (AcendTM 500; Bruker, Biospin international AG, Switzerland) at 100-MHz, 25 °C. Tetramethylsilane (MeSi) was used as an internal shift standard.

### 2.7. Bacterial Identification

For bacterial identification, the genomic DNA of the isolated bacteria was extracted using a DNA extraction kit (GF-1 extraction kit), according to the manufacturer’s instructions. The target gene (16S rRNA) for the identification was amplified using a Polymerase chain reaction with 27F (5′-AGAGTTTGATCMTGGCTCAG-3′) and 1492R (5′-TACGGYTACCTTGTTACGACTT-3′) as forward and reverse primers, respectively. The amplification was performed in 50 μL reaction mixture comprising 25 µL master mix (dNTP, MgCl_2_, Taq polymerase, and buffer), 2 µL each of template DNA, forward and reverse primers, and 19 µL of Ultrapure water [29]. The sequencing of the amplicons was conducted by MyTACG Bioscience Enterprises, Kuala Lumpur, Malaysia. The sequence data of each isolate were assembled and aligned using ClustalW and subsequently compared with existing sequences of the GenBank database using BLAST Available online: http://www.ncbi.nlm.nih.gov/blast (accessed on 13 November 2021) [30]. The identity of each isolate was inferred based on the percentage sequences similarity with the top hit of the BLAST search result (sequence with significant alignment). The sequences of all the identified organisms were subsequently deposited in the GenBank database, and the accession numbers were respectively assigned for reference.

## 3. Results

### 3.1. Bacterial Isolation and Screening for PHA Production

A total of 62 strains of used transformer oil-degrading bacteria were isolated on the mineral salt agar. These isolates demonstrated their capacity to degrade waste transformer oil by their ability to grow in the medium-containing waste transformer oil as the sole carbon source. Table 1 summarizes the abundance of these organisms in relation to the source from which they were obtained.

The results of the PHA screening using the Nile red staining method revealed that 16 (26%) of the total isolates had well-defined, brightly orange cytoplasmatic inclusions when observed using a fluorescence microscope (Table 2)—signifying the potential PHA producers.

**Figure 1:**
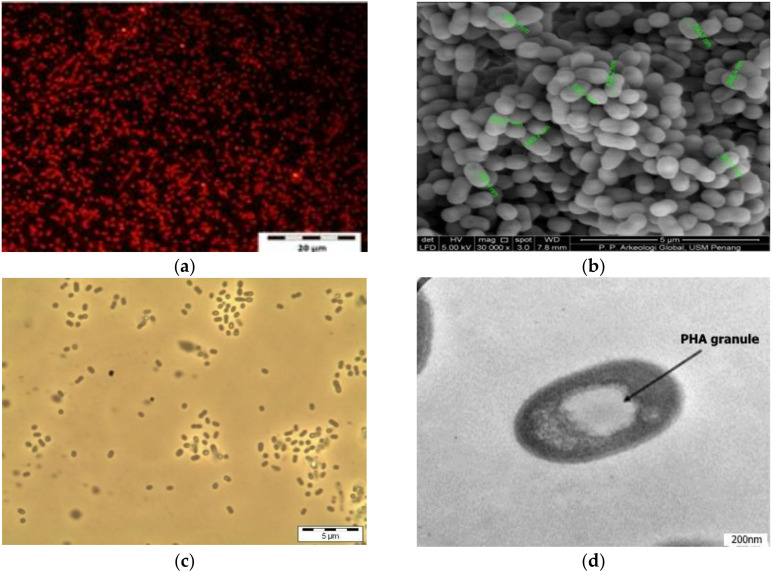
Nile red fluorescence micrograph (**a**), Scanning electron micrograph (**b**), Phase contrast micrograph (**c**), and Transmission electron micrograph (**d**) of a selected isolate SPD2. The black arrow indicates PHA granule within the cell.

Furthermore, highly refractile bodies were detected by phase-contrast microscopy, whereas typical PHA granules within the cells were observed by transmission electron microscopy (TEM), as shown in Figure 1. The results showed that all other samples harbored at least one potential PHA-producing strain, except four samples coded SPA, SJA, BFA, and UTO, which appeared to have none. The wastewater sample collected at Sungai Jelutong had the highest number of potential PHA-producing isolates based on the Nile red screening.

### 3.2. Oil Degradation and PHAs Quantification

The results obtained for the used transformer oil-degrading ability of the bacteria, using solvent extraction and gravimetric method, showed variation among the PHA-producing isolate in this study. The extent of the used transformer oil degradation, PHA accumulation, and cell growth observed among the different isolates is summarized in Table 3.

The PHA biosynthesis was accomplished in shake flasks. The quantitative analysis of the polymer accumulation using the gas chromatographic method revealed that the PHA content ranged from 6.95 ± 0.07 to 33.06 ± 0.19% of CDW. Isolates BFC1 and SPD2 produced a higher percentage of PHA content (>30%) than the other isolates. The lowest PHA content (6.95 ± 0.07%) was recorded from isolate SCA6. The growth measured in terms of cell dry weight shows a range of 0.26 ± 2.25 to 4.82 ± 2.31 g/L, with isolate PBA4 and SJA7 having the lowest and highest, respectively.

### 3.3. PHA Monomer Identification

**Figure 2:**
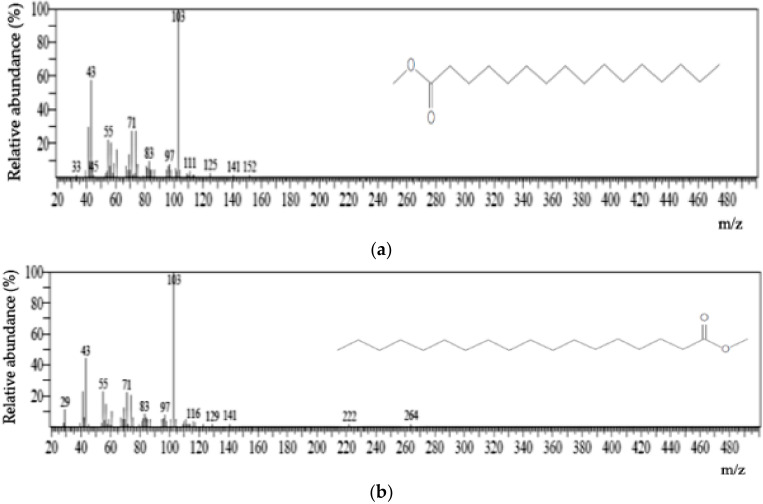
GC ionization mass spectra of the two monomeric components of the PHA produced by the bacterium (*Acinetobacter* sp. Strain AAAID-1.5), (**a**): 3-hydroxyhexanoate methyl ester, (**b**): 3-hydroxyoctanoate methyl ester.

The PHA monomers were identified through GC/MS using computer spectral matching techniques by comparing unknown spectra with the NIST08 Library data. Nuclear magnetic resonance (NMR) was performed for confirmation. The polymer sample extracted from the biomass appeared to have the sticky consistency that is typical of an elastomer. GC/MS ionization mass spectra of the main monomeric components are shown in Figure 2. The analysis revealed that the selected isolates synthesized a polymer whose major monomeric components are 3-hydroxyhexadecanoate (3HHD) and 3-hydroxyoctadecanoate (3HOD).

**Figure 3:**
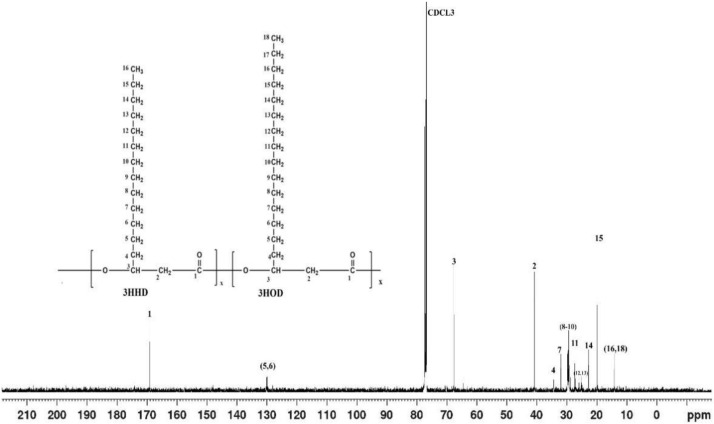
The ^13^C NMR spectrum of the PHA produced by Isolate SPD2 (*Acinetobacter* sp. Strain AAAID-1.5) in a fermentation medium containing waste transformer oil as the sole carbon substrate.

In Figure 3 shown below, the ^13^C NMR frame shift obtained from the NMR analysis of the polymer was presented. From Figure 3, it can be seen that the carbonyl groups (C=O) of the polymer backbone resonated at around 169.14 ppm with no other resonating nearby. This is also comparable to a number of previously reported NMR spectra on PHA characterization [31,32]. The signals up field between 19.76 and 67.62 ppm correspond to methyl, methylene, and methine of the PHA monomers.

### 3.4. Bacterial Identification and Diversity Analysis

All of the 16 potential PHA-producing strains were identified using the molecular method after successful amplification and purification of the target gene (16S rDNA). Table 4 summarizes the details of the bacterial identification.

The bacteria identified belong to four different taxonomic genera of *Acinetobacter*, *Bacillus*, *Proteus*, and *Serratia.*

## 4. Discussion

The isolation of bacteria from the various samples revealed the presence of diverse bacterial strains capable of utilizing used transformer oil as a sole carbon source. The abundance and diversity of the isolated organisms in this research could be linked to the availability of essential nutrients that support microbial life in those environments. The samples collected at Sungai Jelutong and Sungai Pinang had more morphotypes than the other sample sources, with each having eight morphologically distinct colonies. The isolation of these organisms from the various environmental sources was not unexpected. Many microbes that accumulate PHAs as storage materials can be found in natural environments such as soil, seawater, sewage sludge, or compost [33]. There have also been many reports in which diverse species of microbes were isolated from oil-contaminated sites. Specifically, transformer oil-degrading bacteria were previously isolated from oil-contaminated soil. For instance, *Acinetobacter* and *Bacillus* species, in addition to *Pseudomonas* and *Micrococcus,* were isolated from soil samples using mineral salt medium containing used transformer oil as the sole carbon source [34]. Furthermore, the transformer oil is rich in hydrocarbons [35] and those diverse groups of microorganisms, particularly bacteria, were found to have the capacity to degrade these compounds [36].

With respect to the PHAs screening, the results shown in Table 2 revealed that 16 isolates out of the 62 were positive to Nile red fluorescence microscopy. These findings signify that about 26% of all the isolated bacteria are potential PHA producers capable of utilizing the used transformer oil as a carbon source. Several researchers have employed this screening method for the preliminary screening of PHA-producing bacteria to discriminate between PHA-producing strains from the non-producing ones [23,37,38,39,40,41]. Despite its drawback, the method remains a powerful means of discriminating the PHA-positive from the PHA-negative strains, especially when complemented with certain advanced microscopic techniques, such as the use of a transmission electron microscopy that could identify the PHA producers with reasonable certainty. The results of the screening demonstrated the presence of promising, wild-type PHA producers. Interestingly, these findings are comparable to many reports in the literature that reported the identification of potent PHA producers using a similar approach [8,42,43,44].

The extent of the used oil degradation among the bacteria, as shown in Table 3, ranged from 19.58 to 57.51%. The isolates SJB2 were found to degrade the waste oil better than the other isolates; the lowest percentage degradation (19.58 ± 1.73%) was recorded from isolate PBA4. Microorganisms can only degrade oil substrate if they are able to synthesize hydrolytic enzymes, specifically lipases [45]. The efficiency with which an organism expresses the gene coding for these hydrolytic enzymes, and the ability to release the enzymes to their surroundings, could impact the organisms’ oil-degrading capacity. Used transformer oil is a complex mixture of hydrocarbon. Some microorganisms in nature have been found to have the capacity of degrading complex hydrocarbon, either as consortia or single species culture [46]. Biodegradation of waste oils can be considered a natural process limited by several physical and chemical factors [47]. The ability of the isolated bacteria to utilize the used transformer oil, as observed in this work, could be attributed to the possession of hydrolytic enzymes, such as lipases that help in cleavage the complex carbon structures of the oil. The PHA accumulation was quantified using GC analysis; previously, the monomeric components of the PHA were identified through GC/MS, in which the spectra obtained revealed the presence of 3-hydroxyhexadecanoate (3HHD) and 3-hydroxyoctadecanoate (3HOD) as the major polymer constituent. The PHA contents of the isolated bacteria were relatively low, except for isolate BFC1 and SPD2 that produced greater than 30%. This indicates that some organisms are more efficient in bioconversion of the carbon source into the target product (PHA), probably due to their superior metabolic versatility. Furthermore, considering the complexity of various carbon structures associated with the used transformer oil, the organisms have demonstrated a reasonable potential in bioconversion of this used oil. PHA biosynthesis using waste streams as carbon substrates is desirable because such wastes are inexpensive materials that can be obtained with little or no cost. Additionally, utilizing waste as a substrate helps reduce the treatment and management costs. The accumulation of PHA with monomers having up to 16 and 18 carbon atoms represents one of the interesting findings of this research. PHAs with such monomers are rarely encountered. However, there are few studies that documented the detection of such monomers in microbially-synthesized PHAs. Example of such studies is the one in which 3-hydroxyhexadecanoate (3HHD) was found as a constituent of the PHA produced by *Pseudomonas aeruginosa* 42A2 using glucose as a carbon source [34]. Thus, the results of the present work have further substantiated the previous report on the biosynthesis of these unique PHAs. The utilization of used transformer oil as a sole carbon source by the isolated organisms indicated the prospect of WTO utilization in PHA biosynthesis. Free fatty acids (FFAs) are released when oils are hydrolyzed, The FFAs could undergo *β*-oxidation to form a precursor for PHA biosynthesis [48]. Many bacterial species were reported to have the capacity of synthesizing PHA from unrelated carbon feedstock [44]. In another study, *Pseudomonas putida* KT2440 produced medium-chain length PHAs during nutrient limitation when grown on unrelated carbon sources [45]. The application of different waste materials for biosynthesis of PHA is considered a good strategy partly because it is cost-efficient and helps address disposal problems [49,50].

From the results of bacterial identification, it can be observed that *Acinetobacter* species were the most frequently isolated organisms (Table 4). The identity of these isolates was inferred based on their 16S rDNA sequences similarity with the existing sequences of the GenBank of the National Centre for Biotechnology Institute (NCBI). The findings showed that most isolates shared >99% similarities to various bacterial species recovered from similar environmental sites. However, an exception was observed in isolates SCA6, SJB2, SPD4, and SPA6, with a percentage similarity of 81.74, 98.03, 95.78, and 93.00, respectively (Table 4). The soil and wastewater are reservoirs of diverse organisms. These habitats represent an environment where microbes are specifically stimulated to rapidly evolve different metabolic capabilities while competing among themselves and many different species, partly because many essential growth factors often could not be continuously found. Interestingly, the organisms isolated and identified from these environments have demonstrated the ability to actively degrade used transformer oil in the shake flask fermentation resulting in the accumulation of the polyhydroxyalkanoates.

## 5. Conclusions

In conclusion, this research has established the presence of a diverse group of bacteria capable of utilizing used transformer oil as a carbon source to produce polyhydroxyalkanoates, thereby providing the basis for exploring the potentials of used transformer oil as a cheaper alternative carbon feedstock to produce bioplastic.

## Figures and Tables

**Table 1:** The abundance of used transformer oil-degrading bacteria in the various environmental samples collected in Penang.

Sample Code	Sample Type	Sampling Site	GPS		Number of MDC
			Lat.	Longt.	
PBA	Wastewater	Pulau Burung	5°11′53″	100°25′40″	5
PBB	Wastewater	Pulau Burung	5°11′31″	100°25′88″	3
SCA	Wastewater	Sungai Chenaam	5°08′31″	100°24′12″	6
SCC	Sediment	Sungai Chenaam	5°08′31″	100°24′12″	3
SPA	Wastewater	Sungai Pinang	5°08′58″	100°24′38″	8
SPD	Soil	Sungai Pinang	5°08′57″	100°24′38″	5
SJA	Wastewater	Sungai Jelutong	5°24′24″	100°18′50″	8
SJB	Wastewater	Sungai Jelutong	5°24′24″	100°18′50″	5
BFA	Water	Batu Ferringi Beach	5°24′45″	100°20′23″	2
BFC	Sediment	Batu Ferringi Beach	5°24′45″	100°20′23″	6
PPB	Water	Penang Bridge	5°21′28″	100°18′58″	3
PPD	Soil	Penang Bridge	5°21′28″	100°18′58″	5
UTO	Used Oil	TNBR Malaysia	NA	NA	3
					62

Key: MDC = Morphologically distinct colonies, NA = Not applicable.

**Table 2:** Nile red PHA screening response of the used transformer oil-degrading bacteria.

Sample Code	Sample Type	Number of Isolates Screened	Number of Positive Isolates
PBA	Wastewater	5	1
PBB	Wastewater	3	1
SCA	Wastewater	6	2
SCC	Sediment	3	1
SPA	Wastewater	8	0
SPD	Soil	5	3
SJA	Wastewater	8	0
SJB	Wastewater	5	4
BFA	Water	2	0
BFC	Sediment	6	2
PPB	Water	3	1
PPD	Soil	5	1
UTO	Used Oil	3	0
		62	16 (26%)

**Table 3:** The UTO degradation and PHA content of the isolated bacteria.

Isolate Code	Oil Degradation (%)	PHA Content (%)	CDW(g/L)
BFC1	23.78 ± 0.46	33.06 ± 0.19	2.22 ± 0.29
BFC4	33.21 ± 1.96	17.62 ± 0.24	3.49 ± 0.98
PBA4	19.58 ± 1.73	13.75 ± 0.15	2.27 ± 0.24
PBB2	53.65 ± 0.29	8.15 ± 0.07	1.41 ± 1.10
PPD5	52.92 ± 2.35	16.39 ± 0.42	2.64 ± 0.13
SCA1	32.15 ± 0.29	21.33 ± 0.19	0.26 ± 2.25
SCA6	35.12 ± 0.29	6.95 ± 0.07	3.22 ± 0.71
SCC1	53.46 ± 0.29	19.77 ± 0.17	2.29 ± 0.22
SJA7	54.29 ± 0.29	14.42 ± 0.32	4.82 ± 2.31
SJB1	48.08 ± 0.29	18.41 ± 0.62	0.31 ± 2.20
SJB2	57.51 ± 2.06	12.31 ± 0.14	3.91 ± 1.40
SJB3	30.05 ± 1.75	11.13 ± 0.13	1.70 ± 0.81
SB5	46.96 ± 1.62	20.88 ± 0.06	3.91 ± 1.40
SPD2	45.42 ± 1.09	31.12 ± 0.12	4.19 ± 1.68
SPD3	27.12 ± 1.14	21.51 ± 0.17	1.12 ± 1.39
SPD4	28.58 ± 1.20	12.32 ± 0.07	2.37 ± 0.14

Key: CDW = Cell dry weight. a: The data values are the average of the three experimental replicates.

**Table 4:** Identity of PHA-producing bacteria based on 16S rDNA analysis.

IsolateCode	Top Hit of the NCBI BLAST Search	Similarity (%)	Organism Identified	GenBank Accession Number
BFC1	*Acinetobacter* sp. Strain Gamma-15	99.45	*Acinetobacter* sp.	MZ411707
BFC4	*Acinetobacter* sp. Strain WWW203	99.01	*Acinetobacter* sp.	MZ411708
PBA4	Bacterium Stain Glm4	99.79	*Acinetobacter* sp.	MZ411699
PBB2	*Serratia marcescens* Strain KS10	99.79	*Serratia marcescens*	MZ411696
PPD5	*Acinetobacter* sp. HS-B1	99.51	*Acinetobacter* sp.	MZ411697
SCA1	Bacterium Stain CH-12	99.38	*Acinetobacter* sp.	MZ411704
SCA6	*Proteus* Vulgaris	81.74	*Proteus* sp.	MZ411698
SCC1	*Acinetobacter* sp. Strain PrPc065	99.86	*Acinetobacter* sp.	MZ411695
SJA7	Uncultured Bacterium Clone SH201207-62	99.65	*Acinetobacter* sp.	MZ411705
SJB1	*Serratia marcescens* Strain RS	99.93	*Serratia marcescens*	MZ411693
SJB2	Bacterium Stain N13.7	98.03	*Acinetobacter* sp.	MZ411694
SJB3	*Serratia marcescens* Strain KS10	99.51	*Serratia marcescens*	MZ411702
SJB5	*Acinetobacter bereziniae* Strain XH901	99.72	*Acinetobacter* sp.	MZ411703
SPD2	*Acinetobacter* sp. Strain PrPc065	99.44	*Acinetobacter* sp.	MZ411700
SPD3	Uncultured Gamma Proteobacterium Clone FTL260	99.72	*Serratia* sp.	MZ411706
SPD4	*Bacillus aryabhattai* Strain WH6	95.78	*Bacillus* sp.	MZ411701

## Data Availability

Not applicable.

## References

[1] Możejko-Ciesielska J., Kiewisz R. (2016). Bacterial polyhydroxyalkanoates: Still fabulous?. Microbiol. Res..

[2] Chanprateep S. (2010). Current trends in biodegradable polyhydroxyalkanoates. J. Biosci. Bioeng..

[3] Lopez G., Artetxe M., Amutio M., Alvarez J., Bilbao J., Olazar M. (2018). Recent advances in the gasification of waste plastics. A critical overview. Renew. Sustain. Energy Rev..

[4] Prasanna Raj Yadav S., Saravanan C.G., Vallinayagam R., Vedharaj S., Roberts W.L. (2015). Fuel and engine characterization study of catalytically cracked waste transformer oil. Energy Convers. Manag..

[5] Koller M. (2018). Biodegradable and biocompatible polyhydroxy-alkanoates (PHA): Auspicious microbial macromolecules for pharmaceutical and therapeutic applications. Molecules.

[6] Kunasundari B., Sudesh K. (2011). Isolation and recovery of microbial polyhydroxyalkanoates. Express Polym. Lett..

[7] Cavalheiro J.M.B.T., de Almeida M.C.M.D., Grandfils C., da Fonseca M.M.R. (2009). Poly(3-hydroxybutyrate) production by Cupriavidus necator using waste glycerol. Process Biochem..

[8] Teeka J., Imai T., Cheng X., Reungsang A., Higu T., Yamamoto K., Sekine M. (2010). Screening of PHA-Producing Bacteria Using Biodiesel-Derived Waste Glycerol as a Sole Carbon Source. J. Water Environ. Technol..

[9] Loo C.Y., Lee W.H., Tsuge T., Doi Y., Sudesh K. (2005). Biosynthesis and characterization of poly (3-hydroxybutyrate-co-3- hydroxyhexanoate) from palm oil products in a Wautersia eutropha mutant. Biotechnol. Lett..

[10] Posada J.A., Naranjo J.M., López J.A., Higuita J.C., Cardona C.A. (2011). Design and analysis of poly-3-hydroxybutyrate production processes from crude glycerol. Process Biochem..

[11] Teeka J., Imai T., Reungsang A., Cheng X., Yuliani E., Thiantanankul J., Poomipuk N., Yamaguchi J., Jeenanong A., Higuchi T. (2012). Characterization of polyhydroxyalkanoates (PHAs) biosynthesis by isolated Novosphingobium sp. THA-AIK7 using crude glycerol. J. Ind. Microbiol. Biotechnol..

[12] Fukui T., Doi Y. (1998). Efficient production of polyhydroxyalkanoates from plant oils by Alcaligenes eutrophus and its recombinant strain. Appl. Microbiol. Biotechnol..

[13] Cerrone F., Choudhari S.K., Davis R., Cysneiros D., O’Flaherty V., Duane G., Casey E., Guzik M.W., Kenny S.T., Babu R.P. (2014). Medium chain length polyhydroxyalkanoate (mcl-PHA) production from volatile fatty acids derived from the anaerobic digestion of grass. Appl. Microbiol. Biotechnol..

[14] Chen Z., Huang L., Wen Q., Guo Z. (2015). Efficient polyhydroxyalkanoate (PHA) accumulation by a new continuous feeding mode in three-stage mixed microbial culture (MMC) PHA production process. J. Biotechnol..

[15] Pozo C., Martínez-Toledo M.V., Rodelas B., González-López J. (2002). Effects of culture conditions on the production of polyhydroxyalkanoates by Azotobacter chroococcum H23 in media containing a high concentration of alpechín (wastewater from olive oil mills) as primary carbon source. J. Biotechnol..

[16] Griffin G.J.L. (1995). Chemistry and Technology of Biodegradable Polymers.

[17] Philip S., Keshavarz T., Roy I. (2007). Polyhydroxyalkanoates: Biodegradable polymers with a range of applications. J. Chem. Technol. Biotechnol..

[18] Keshavarz T., Roy I. (2010). Polyhydroxyalkanoates: Bioplastics with a green agenda. Curr. Opin. Microbiol..

[19] Chee J., Yoga S., Lau N., Ling S., Abed R.M.M. (2010). Bacterially Produced Polyhydroxyalkanoate (PHA): Converting Renewable Resources into Bioplastics. Curr. Res. Technol. Educ. Top. Appl. Microbiol. Microb. Biotechnol..

[20] Pradeep J.A., Kishore Kumar K., Scholars U., Balasubramanian D. (2016). Biofuel Production Using Butanol and Used Transformer Oil. Am. J. Eng. Res..

[21] Bahera P. (2013). Experimental Studies on Utilization of Used Transformer Oil as an Alternative Fuel in a DI Diesel Engine. Ph.D. Thesis.

[22] Amirul A.A., Yahya A.R.M., Sudesh K., Azizan M.N.M., Majid M.I.A. (2008). Biosynthesis of poly(3-hydroxybutyrate-co-4-hydroxybutyrate) copolymer by Cupriavidus sp. USMAA1020 isolated from Lake Kulim, Malaysia. Bioresour. Technol..

[23] Spiekermann P., Rehm B.H.A., Kalscheuer R., Baumeister D., Steinbüchel A. (1999). A sensitive, viable colony staining method using Nile red for direct screening of bacteria that accumulate polyhydroxyalkanoic acids and other lipid storage compounds. Arch. Microbiol..

[24] López-Cortés A., Lanz-Landázuri A., García-Maldonado J.Q. (2008). Screening and isolation of PHB-producing bacteria in a polluted marine microbial mat. Microb. Ecol..

[25] Fernández D., Rodríguez E., Bassas M., Viñas M., Solanas A.M., Llorens J., Marqués A.M., Manresa A. (2005). Agro-industrial oily wastes as substrates for PHA production by the new strain Pseudomonas aeruginosa NCIB 40045: Effect of culture conditions. Biochem. Eng. J..

[26] Jayashree R., Nithya S.E., Prasanna P.R., Krishnaraju M. (2012). Biodegradation capability of bacterial species isolated from oil contaminated soil. J. Acad. Ind. Res..

[27] Huong K.H., Teh C.H., Amirul A.A. (2017). Microbial-based synthesis of highly elastomeric biodegradable poly(3-hydroxybutyrate-co-4-hydroxybutyrate) thermoplastic. Int. J. Biol. Macromol..

[28] Guo W., Duan J., Geng W., Feng J., Wang S., Song C. (2013). Comparison of medium-chain-length polyhydroxyalkanoates synthases from Pseudomonas mendocina NK-01 with the same substrate specificity. Microbiol. Res..

[29] Turner S., Pryer K.M., Miao V.P.W., Palmer J.D. (1999). Investigating deep phylogenetic relationships among cyanobacteria and plastids by small subunit rRNA sequence analysis. J. Eukaryot. Microbiol..

[30] Rédei G.P. (2008). CLUSTAL W: Improving the sensitivity of progressive multiple sequence alignment through sequence weighting, position-specific gap penalties and weight matrix choice. Encycl. Genet. Genom. Proteom. Inform..

[31] Follonier S., Riesen R., Zinn M. (2015). Pilot-scale production of functionalized mcl-PHA from grape pomace supplemented with fatty acids. Chem. Biochem. Eng. Q..

[32] Wang H.H., Li X.T., Chen G.Q. (2009). Production and characterization of homopolymer polyhydroxyheptanoate (P3HHp) by a fadBA knockout mutant Pseudomonas putida KTOY06 derived from P. putida KT2442. Process Biochem..

[33] Gmbh C.L., Industries A., Gmbh C.L., Industries A. (1992). Industrial production of poly-/3-hydroxybutyrate. FEMS Microbiol. Rev..

[34] Safi A., Subhanullah K., Ayaz M., Attaullah, Khatak B., Akbar N., Khan I., Asif M., Khan N., Ullah S. (2015). Isolation and Identification of Bacteria from Transformer Oil Contaminated Soil. Br. Microbiol. Res. J..

[35] Ayandele A., Fagade O. (2012). Effect of ammonium salts on the biodegradation of used transformer oil using locally isolated microorganisms. Agric. Biol. J. North Am..

[36] Sobiecka E., Cedzynska K., Bielski C., Antizar-Ladislao B. (2009). Biological treatment of transformer oil using commercial mixtures of microorganisms. Int. Biodeterior. Biodegrad..

[37] Amirul A.A., Syairah S.N., Yahya A.R.M., Azizan M.N.M., Majid M.I.A. (2008). Synthesis of biodegradable polyesters by Gram negative bacterium isolated from Malaysian environment. World J. Microbiol. Biotechnol..

[38] Kung S.S., Chuang Y.C., Chen C.H., Chien C.C. (2007). Isolation of polyhydroxyalkanoates-producing bacteria using a combination of phenotypic and genotypic approach. Lett. Appl. Microbiol..

[39] Mravec F., Obruca S., Krzyzanek V., Sedlacek P., Hrubanova K., Samek O., Kucera D., Benesova P., Nebesarova J. (2016). Accumulation of PHA granules in Cupriavidus necator as seen by confocal fluorescence microscopy. FEMS Microbiol. Lett..

[40] Redzwan G., Gan S., Tan I.K.P. (1997). Short Communication: Isolation of polyhydroxyalkanoate-producing bacteria from an integrated-farming pond and palm-oil mill ef ¯ uent ponds. World J. Microbiol. Biotechnol..

[41] Tyo K.E., Zhou H., Stephanopoulos G.N. (2006). High-throughput screen for poly-3-hydroxybutyrate in *Escherichia coli* and *Synechocystis* sp. strain PCC6803. Appl. Environ. Microbiol..

[42] Chaudhry W.N., Jamil N., Ali I., Ayaz M.H., Hasnain S. (2011). Screening for polyhydroxyalkanoate (PHA)-producing bacterial strains and comparison of PHA production from various inexpensive carbon sources. Ann. Microbiol..

[43] Motamedi H., Ardakani M.R., Mayeli N. (2015). Isolation and screening of native polyhydroxyalkanoate producing bacteria from oil contaminated soils of Abadan refinery. Biol. J. Microorg..

[44] Tan W.A., Wijaya I., Purwadaria T. (2019). Bioprospecting of polyhydroxyalkanoates-producing bacteria from Indonesian marine environment. Biodiversitas.

[45] Cammarota M.C., Freire D.M.G. (2006). A review on hydrolytic enzymes in the treatment of wastewater with high oil and grease content. Bioresour. Technol..

[46] Ghazali F.M., Rahman R.N.Z.A., Salleh A.B., Basri M. (2004). Biodegradation of hydrocarbons in soil by microbial consortium. Int. Biodeterior. Biodegrad..

[47] Chandra S., Sharma R., Singh K., Sharma A. (2013). Application of bioremediation technology in the environment contaminated with petroleum hydrocarbon. Ann. Microbiol..

[48] Ashby R.D., Solaiman D.K.Y., Foglia T.A. (2004). Bacterial poly(hydroxyalkanoate) polymer production from the biodiesel co-product stream. J. Polym. Environ..

[49] Koller M. (2018). A review on established and emerging fermentation schemes for microbial production of polyhydroxyalkanoate (PHA) biopolyesters. Fermentation.

[50] Koller M., Bona R., Braunegg G., Hermann C., Horvat P., Kroutil M., Martinz J., Neto J., Pereira L., Varila P. (2005). Production of polyhydroxyalkanoates from agricultural waste and surplus materials. Biomacromolecules.

